# Association between antipsychotic use and acute ischemic heart disease in women but not in men: a retrospective cohort study of over one million primary care patients

**DOI:** 10.1186/s12916-020-01765-w

**Published:** 2020-11-02

**Authors:** Francisco T. T. Lai, Bruce Guthrie, Stewart W. Mercer, Daniel J. Smith, Benjamin H. K. Yip, Gary K. K. Chung, Kam-Pui Lee, Roger Y. Chung, Patsy Y. K. Chau, Eliza L. Y. Wong, Eng-Kiong Yeoh, Samuel Y. S. Wong

**Affiliations:** 1 The Jockey Club School of Public Health and Primary Care, Faculty of Medicine, The Chinese University of Hong Kong, Hong Kong SAR, New Territories China; 2 Usher Institute, The University of Edinburgh, Scotland, UK; 3 Institute of Health & Wellbeing, The University of Glasgow, Scotland, UK

**Keywords:** Cardiovascular diseases, Community psychiatry, Myocardial infarction, Primary health care, Psychotic disorders, Schizophrenia

## Abstract

**Background:**

Research comparing sex differences in the effects of antipsychotic medications on acute ischemic heart disease (IHD) is limited and the findings ambiguous. This study aimed to investigate these associations within a primary care setting.

**Methods:**

Hong Kong public general outpatient electronic records of patients aged 45+ during 2007–2010 were extracted, with the last consultation date as the baseline for a 4-year follow-up period to observe acute IHD hospitalizations (2011–2014). Antipsychotic use was defined as any prescription over the previous 12 months from a list of 16 antipsychotics, while acute IHD was defined by ICD-9: 410.00–411.89. Both sex-specific and sex-combined (both sexes) mixed-effects Cox models (random intercept across 74 clinics) were implemented to examine the association and test the interaction between antipsychotics and sex.

**Results:**

Among 1,043,236 included patients, 17,780 (1.7%) were prescribed antipsychotics, and 8342 (0.8%) developed IHD. In sex-specific analyses, antipsychotic prescription was associated with a 32% increased hazard rate of acute IHD among women (95% CI 1.05–1.67) but not among men. A likelihood ratio test comparing sex-combined models with and without the interaction between antipsychotic use and sex suggested significant interaction (*χ*^2^ = 4.72, *P* = 0.030). The association between antipsychotic use and IHD among women attenuated and became non-significant when haloperidol was omitted from the operationalization of antipsychotic use (HR = 1.23, 95% CI 0.95–1.60).

**Conclusion:**

Our results suggest that antipsychotic prescription is moderately associated with an increased risk of acute IHD among women in primary care and this relationship may be explained by specific antipsychotics. Further research should observe and capture the potential intermediary mechanisms and the dose-response relationship of this association to provide more rigorous evidence to establish causality and inform clinical practices.

## Background

Antipsychotic medications commonly prescribed for patients with a variety of mental disorders such as schizophrenia, bipolar disorder, and dementia are generally believed to entail a heightened risk of acute ischemic heart disease (IHD) [1]. However, meta-analyses have reported conflicting results on this association [2–5]. For instance, in a review of 399,868 participants from nine observational studies [2], antipsychotic medications were found to be associated with 121%-elevated odds of myocardial infarction, whereas in another meta-analysis [3], no significant association was observed. These mixed findings may be due to a heterogeneity across study populations [4], suggesting that this association may only be present in populations with certain characteristics.

Several intermediary mechanisms have been proposed in support of the association, for example, the higher prevalence of metabolic abnormalities among patients on antipsychotic medications [6] and the blockade of specific neurotransmitter receptors which has been identified to be related to IHD risks [7]. Although it is difficult to observe and incorporate all potential mediators to examine the relative importance of the mechanisms, the comparison of the presence or absence, as well as the varying strength, of this association between specific subpopulations or sociodemographic strata may inform the inquiry about the potential underlying mechanisms considerably. This is because some mechanisms may operate more strongly in certain subpopulations, indicated by stronger statistical associations.

Sex difference in the association between antipsychotics and IHD, in particular, has been little examined in previous research, despite the growing evidence of potentially different physiological mechanisms in the development of cardiovascular diseases [8] and different prevalence of tobacco [9] and alcohol use [10] as well as sedentary lifestyle [11] between men and women. There is also a lack of large-scale studies in primary care populations, whose needs typically arouse much less medical attention because of the seemingly milder risks compared with inpatients who have already experienced an acute episode [12].

In this study, we used a large multicenter public primary care database of over one million middle-aged and older patients in Hong Kong to examine the association between antipsychotics and acute IHD incidence and tested for the potential sex difference of this association.

## Methods

### Study design and sample selection

We adopted a retrospective cohort design for this investigation which was under a broader project on health care for older people commissioned by the Hong Kong Government, and were allowed access to the clinical records of patients aged 45 or above from the public health care sector. The Hospital Authority (HA), a major provider of public outpatient services and the sole provider of public inpatient services in Hong Kong, provided data for the analysis. We examined the records of all patients aged 45 or more who visited any of the 74 general outpatient clinics run by the HA during January 1, 2007, to December 31, 2010, for data extraction to form the closed cohort.

We took the last visit by these patients during the period as the baseline and retrieved the corresponding outpatient clinical records over the 12 months prior to the baseline (including baseline visit records). We excluded patients with an existing diagnosis of IHD (International Classification of Primary Care (ICPC) codes K74, K75, and K76) or any previous admission to public hospitals to enable the results to better inform primary care practice for those who had not presented with recent acute health problems or with an existing IHD diagnosis, who typically received less medical attention.

The patients were then followed up until they were admitted to any public hospitals through the Accident and Emergency Unit due to IHD or any other reasons, or until 4 years after the baseline (January 2011–December 2014).

The authors assert that all procedures contributing to this work comply with the ethical standards of the relevant national and institutional committees on human experimentation and with the Helsinki Declaration of 1975, as revised in 2008. All procedures involving human subjects/patients were approved by the Survey and Behavioral Ethics Committee of the Chinese University of Hong Kong (dated 25 August 2015, Project Code: Elderly Care – CUHK). As only secondary analysis of the anonymized unidentifiable patient records was involved, no written consent was required.

### Study outcome—time to acute hospitalization due to IHD

The time duration from the baseline to the first hospital admission through the Accident and Emergency Unit with a primary diagnosis of IHD (defined by International Classification of Diseases, Ninth Revision (ICD-9)) was used as the outcome of the analysis. The ICD-9 codes used to identify acute IHD ranged from 410.00 to 411.89 (down to two decimal places when applicable).

### Exposure—antipsychotic use

Antipsychotic use was the main exposure in the analysis. It was defined by any prescriptions of the following 16 antipsychotic medications (generic drug name) over the 12 months prior to the baseline: amisulpride, aripiprazole, chlorpromazine, clozapine, fluphenazine, haloperidol, olanzapine, paliperidone, perphenazine, quetiapine, risperidone, sulpiride, thioridazine, thiothixene, trifluoperazine, and ziprasidone.

### Effect modifier—biological sex

Biological sex (women as referent) was tested as an effect modifier of the association between antipsychotics on time to acute IHD hospitalization.

### Multivariable adjustment

We included other baseline variables (according to the records over the 12 months prior to the baseline) to adjust for their potential confounding effects. First, baseline morbidities diagnosed by primary care physicians including schizophrenia (ICPC code P72), dementia (ICPC code P70), depression (ICPC codes P03, P76, and P77), bipolar disorder (ICPC code P73), diabetes (ICPC codes T89 and T90), hypertension (ICPC codes K86 and K87), atrial fibrillation (ICPC code K78), stroke (ICPC codes K89 and K90), and lipid disorder (ICPC code T93) were adopted. Second, we further included tobacco abuse (ICPC code P17) to proxy the unobserved smoking status. Third, we included the prescription of any antidepressants (amitriptyline, clomipramine, citalopram, dosulepin, doxepin, fluoxetine, imipramine, lofepramine, mirtazapine, moclobemide, nortriptyline, paroxetine, phenelzine, reboxetine, sertraline, tranylcypromine, trazodone, venlafaxine) over the past year as antidepressants have been shown to be related to IHD and are sometimes used in combination with antipsychotics [13, 14]. Fourth, we included the prescription of statin (atorvastatin, cerivastatin, fluvastatin, lovastatin, pravastatin, pitavastatin, simvastatin, rosuvastatin) as a dichotomized variable (prescribed or not prescribed over the 12 months) and specified an interaction between lipid disorder and statin prescription.

### Statistical analysis

We ran a mixed-effects Cox model (with random intercept across the 74 general outpatient clinics) to examine the hazard ratios of acute hospitalization due to IHD between those prescribed antipsychotics and those who were not, with potential confounders adjusted (both with sex-stratified and with sex-combined samples). Based on this model, we further constructed an extended model in which the interaction between antipsychotics and sex was specified. Likelihood ratio test was used to test for this interaction.

We conducted multiple sets of sensitivity analyses to test for the robustness of the results. First, we adopted the leave-one-out approach to replicate the main analysis multiple times in each of which we removed one of the antipsychotic medications in the definition of antipsychotic use. Likewise, we used a similar approach to replicate the analysis repeatedly with patients admitted for each subtype of acute IHD (ICD-9: 410.00–411.89) excluded. These replications could reveal the potential changes in the results with alternative operationalization of the outcome and main exposure, respectively. Motivated by the results of the leave-one-out analysis on specific antipsychotic medications, we further replicated the analysis with antipsychotic use replaced by only haloperidol prescription as main exposure, with the prescription of other antipsychotics adjusted, for a comparison. We then replicated the analysis with the sample restricted to only those prescribed antipsychotics and examined the association of each antipsychotic with acute IHD. Second, we varied the threshold of the number of antipsychotic medications for the operationalization of antipsychotic use from one to eight for another set of replicated analyses. Third, we replicated the main analysis on a larger sample selected without applying the exclusion criteria of previous hospital admission and IHD diagnosis to examine the impact of those exclusion criteria on the results. Fourth, the main analysis was replicated without including the preexisting diagnoses of diabetes, hypertension, and lipid disorder (and statin prescription) which may potentially act as the intermediary mechanisms between antipsychotic use and IHD.

The mixed-effects Cox model was implemented using the “coxme” package [15] in R, version 3.6.0 (R Foundation for Statistical Computing, Vienna, Austria). Two patients with incomplete data were excluded from the analysis.

## Results

### Descriptive results

**Fig. 1:**
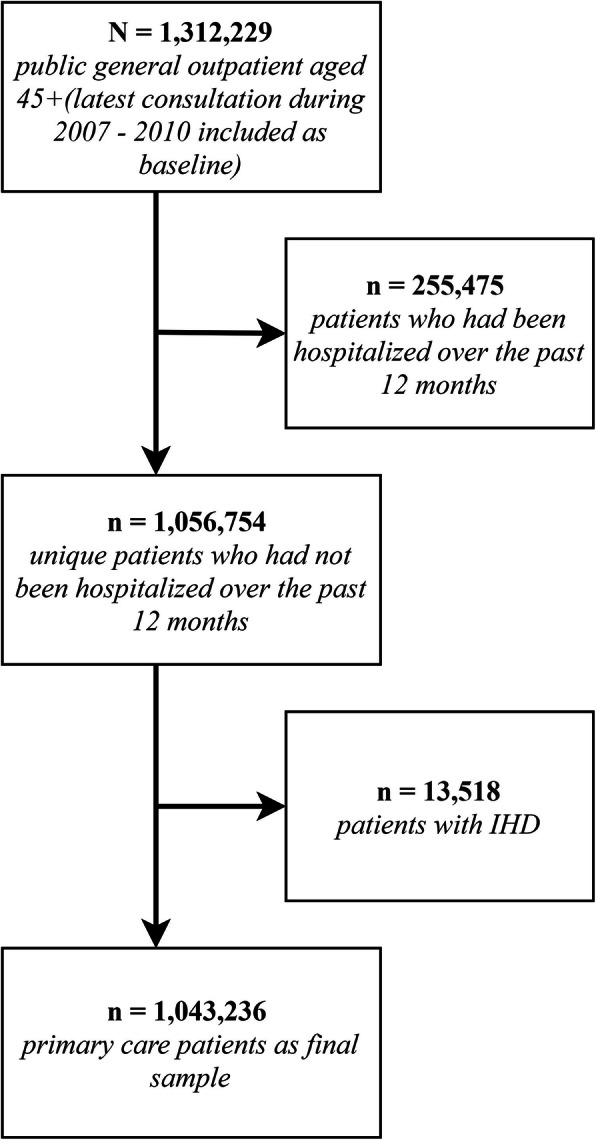
Flow chart showing the procedures of the sample selection for this study

During January 1, 2007, to December 31, 2010, a total of 15,054,785 general outpatient clinic visits by 1,312,229 patients aged 45 or above were recorded. We removed 255,475 patients with hospital admission history over the past 12 months, then further excluded 13,518 having the diagnoses of IHD. Eventually, 1,043,236 primary care patients were included for analysis. The mean (standard deviation) follow-up period was 1216.7 (454.7) days. Figure 1 shows the procedures of data selection for this study.

Table 1 shows the descriptive statistics of the cohort. In total, there were 17,780 patients being prescribed antipsychotics and 0.8% of them were admitted due to IHD through the Accident and Emergency Unit within 4 years, compared similarly with those without antipsychotic prescriptions without any multivariable adjustment. Sex ratios between the two groups were similar, with around 40% men. There was a higher prevalence of diabetes (17.8% versus 14.4%, *P* < 0.001) and stroke (2.2% versus 1.2%, *P* < 0.001) but a lower prevalence of hypertension (31.9% versus 36.9%, *P* < 0.001) among those prescribed antipsychotics than those who were not. Both lipid disorder prevalence (7.2% versus 9.9%, *P* < 0.001) and statin prescription rate (6.6% versus 7.6%, *P* < 0.001) were lower among those on antipsychotics. A notably higher rate of antidepressant prescription was observed among those on antipsychotics too (27.6% versus 2.8%, *P* < 0.001).

**Table 1:** Descriptive statistics of the sample (*N* = 1,043,236)

	Without antipsychotic prescriptions	With antipsychotic prescriptions	*P* value*
*n*	1,025,456	17,780	
Sex (%)			< 0.001
Men	436,623 (42.6)	7110 (40.0)	
Women	588,833 (57.4)	10,670 (60.0)	
Age (%)			< 0.001
45–54	341,743 (33.3)	6614 (37.2)	
55–64	306,180 (29.9)	5037 (28.3)	
65–74	200,401 (19.5)	2421 (13.6)	
75–84	138,672 (13.5)	2354 (13.2)	
85+	38,460 (3.8)	1354 (7.6)	
International Classification of Primary Care Diagnoses (%)			
Schizophrenia	69 (0.0)	156 (0.9)	< 0.001
Depression	1845 (0.2)	36 (0.2)	0.539
Bipolar disorder	5 (0.0)	3 (0.0)	< 0.001
Dementia	1338 (0.1)	326 (1.8)	< 0.001
Tobacco abuse	6209 (0.6)	116 (0.7)	0.453
Diabetes	147,949 (14.4)	3160 (17.8)	< 0.001
Hypertension	378,476 (36.9)	5663 (31.9)	< 0.001
Atrial fibrillation	3738 (0.4)	78 (0.4)	0.118
Stroke	12,388 (1.2)	384 (2.2)	< 0.001
Lipid disorder	101,192 (9.9)	1285 (7.2)	< 0.001
Statin prescription (%)	77,410 (7.6)	1169 (6.6)	< 0.001
Antidepressant prescription (%)	29,166 (2.8)	4905 (27.6)	< 0.001
Acute ischemic heart disease hospitalization within 4 years (%)			0.691
Not hospitalized	1,017,251 (99.2)	17,643 (99.2)	
Hospitalized	8205 (0.8)	137 (0.8)	

*P value of chi-square tests/Fisher’s exact tests for the difference between patients on antipsychotics and those who were not

**Fig. 2:**
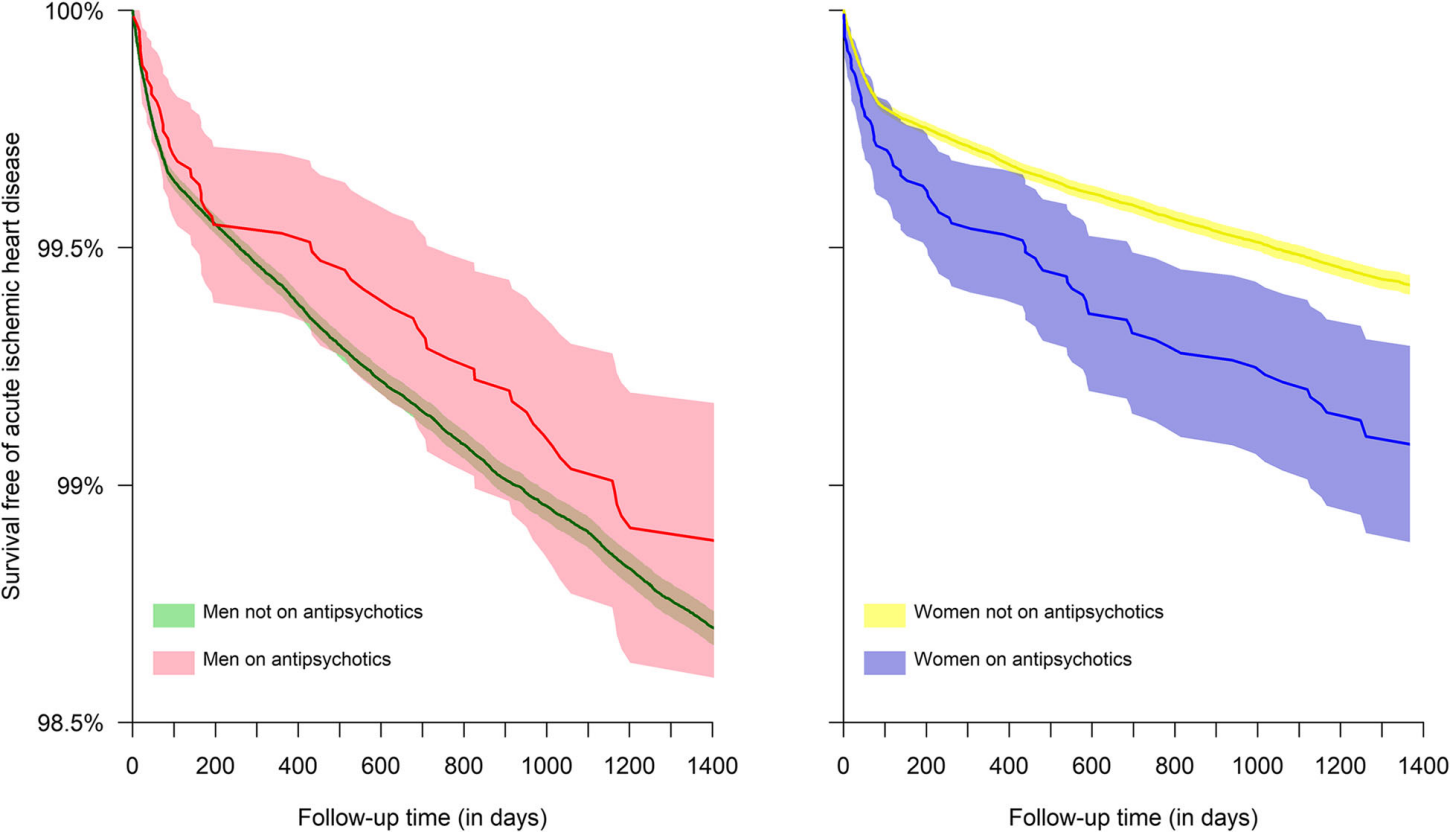
Patterns of survival free of acute hospitalization due to ischemic heart disease by sex and prescription of antipsychotics. Shaded area represents 95% confidence intervals

Figure 2 shows the acute IHD-free survival patterns across follow-up time by sex and by the prescription of antipsychotics. The impact of antipsychotics on the patterns differed drastically by sex. Antipsychotics were associated with a significantly worse survival pattern among women, whereas men on antipsychotics had a similar survival pattern compared with those not prescribed antipsychotics.

### Mixed-effects Cox model

Table 2 shows the results of the mixed-effects Cox regression analysis without specifying the interaction between antipsychotic use and sex. When men and women were separately analyzed (sex-specific models: first and second columns of Table 2), antipsychotics were associated with an increased hazard rate of acute IHD among women (HR = 1.32, 95% CI 1.05–1.67) but not among men. When both men and women were included in the analysis (sex-combined model: third column of Table 2), antipsychotics were associated with acute IHD with a hazard ratio of 1.19 (95% CI 1.00–1.41).

**Table 2:** Adjusted hazard ratios [95% confidence intervals] of acute ischemic heart disease

	Sex-specific models		Sex-combined model
	Men only	Women only	Men + women
***Antipsychotic prescription***	***0.95 [0.73, 1.23]***	1.32 [1.05, 1.67]*	1.19 [1.00, 1.41]*
***Men (women as referent)***			2.19 [2.10, 2.29]***
Age (in 10 years)	1.55 [1.51, 1.59]***	2.37 [2.30, 2.44]***	1.86 [0.55, 6.32]**
Antidepressant prescription	1.14 [0.94, 1.39]	1.15 [0.96, 1.37]	1.16 [1.01, 1.32]*
International Classification of Primary Care Diagnoses as baseline^a^			
Dementia	0.99 [0.47, 2.08]	0.54 [0.30, 0.99]*	0.78 [0.49, 1.23]
Tobacco abuse	1.55 [1.28, 1.88]***	3.16 [1.83, 5.46]***	1.68 [1.40, 2.01]***
Diabetes	1.29 [1.20, 1.38]***	1.62 [1.49, 1.76]***	1.43 [1.35, 1.50]***
Hypertension	1.60 [1.50, 1.70]***	1.50 [1.39, 1.63]***	1.55 [1.47, 1.62]***
Atrial fibrillation	1.20 [0.86, 1.67]	1.06 [0.72, 1.55]	1.16 [0.90, 1.49]
Stroke	1.31 [1.11, 1.55]**	1.49 [1.23, 1.82]***	1.37 [1.20, 1.55]***
Lipid disorder without any statin prescription^b^	1.12 [1.01, 1.24]*	1.15 [1.03, 1.28]*	1.14 [1.06, 1.23]***
Statin prescription for those without lipid disorder^b^	1.62 [1.46, 1.80]***	1.59 [1.38, 1.82]***	1.59 [1.47, 1.73]***
Lipid disorder with statin prescription^b^	0.73 [0.61, 0.87]***	0.63 [0.50, 0.78]***	0.69 [0.60, 0.79]***

***P < 0.001, **P < 0.01, *P < 0.05

^a^Hazard ratio for schizophrenia, depression, and bipolar disorder is not shown because of extremely wide non-significant confidence intervals due to low prevalence

^b^Referent group: those without lipid disorder and without any statin prescription

Table 3 shows the results of the sex-combined model with an additionally estimated interaction between antipsychotic use and sex. The increase of hazard rate of acute IHD associated with antipsychotics was only observed among women (HR = 1.43, 95% CI 1.13–1.79) but not among men (HR = 2.21, 95% CI 2.11–2.31 for those who were not on antipsychotics versus HR = 2.16, 95% CI 1.67–2.80 for those who were prescribed antipsychotics). Likelihood ratio test result suggested significant interaction between antipsychotic use and sex (*χ*^2^ = 4.72, *P* = 0.030).

**Table 3:** Adjusted hazard ratios [95% confidence intervals] of acute ischemic heart disease estimated from a mixed-effects Cox model with the specification of the interaction between antipsychotic use and sex

***Antipsychotic prescription for women***^***a***^	1.43 [1.13, 1.79]**
***Men who were not on antipsychotics***^***a***^	2.21 [2.11, 2.31]***
***Men with antipsychotic prescription***^***a***^	2.16 [1.67, 2.80]***
Age (in 10 years)	1.86 [1.83, 1.90]***
Antidepressant prescription	1.15 [1.01, 1.32]*
International Classification of Primary Care Diagnoses as baseline^b^	
Dementia	0.76 [0.48, 1.22]
Tobacco abuse	1.67 [1.40, 2.01]***
Diabetes	1.43 [1.35, 1.50]***
Hypertension	1.55 [1.47, 1.62]***
Atrial fibrillation	1.16 [0.90, 1.49]
Stroke	1.37 [1.20, 1.55]***
Lipid disorder without any statin prescription^c^	1.14 [1.06, 1.23]***
Statin prescription for those without lipid disorder^c^	1.59 [1.47, 1.73]***
Lipid disorder with statin prescription^c^	0.69 [0.60, 0.79]***

***P < 0.001, **P < 0.01, *P < 0.05

^a^Referent group: women who were not on antipsychotics

^b^Hazard ratio for schizophrenia, depression, and bipolar disorder is not shown because of extremely wide non-significant confidence intervals due to low prevalence

^c^Referent group: those without lipid disorder and without any statin prescription

According to this model (Table 3), antidepressant prescriptions (HR = 1.15, 95% CI 1.01–1.32), tobacco abuse (HR = 1.67, 95% CI 1.40–2.01), diabetes (HR = 1.43, 95% CI 1.35–1.50), hypertension (HR = 1.55, 95% CI 1.47–1.62), stroke (HR = 1.37, 95% CI 1.20–1.55), lipid disorder (HR = 1.14, 95% CI 1.06–1.23), and statin prescription for those without lipid disorder (HR = 1.59, 95% CI 1.47–1.73) were associated with a greater risk of acute IHD, while statin prescription for those with lipid disorder was associated with a reduced risk (HR = 0.69, 95% CI 0.60–0.79). No substantial sex differences were observed in the results regarding the potential confounders.

### Sensitivity analysis

**Fig. 3:**
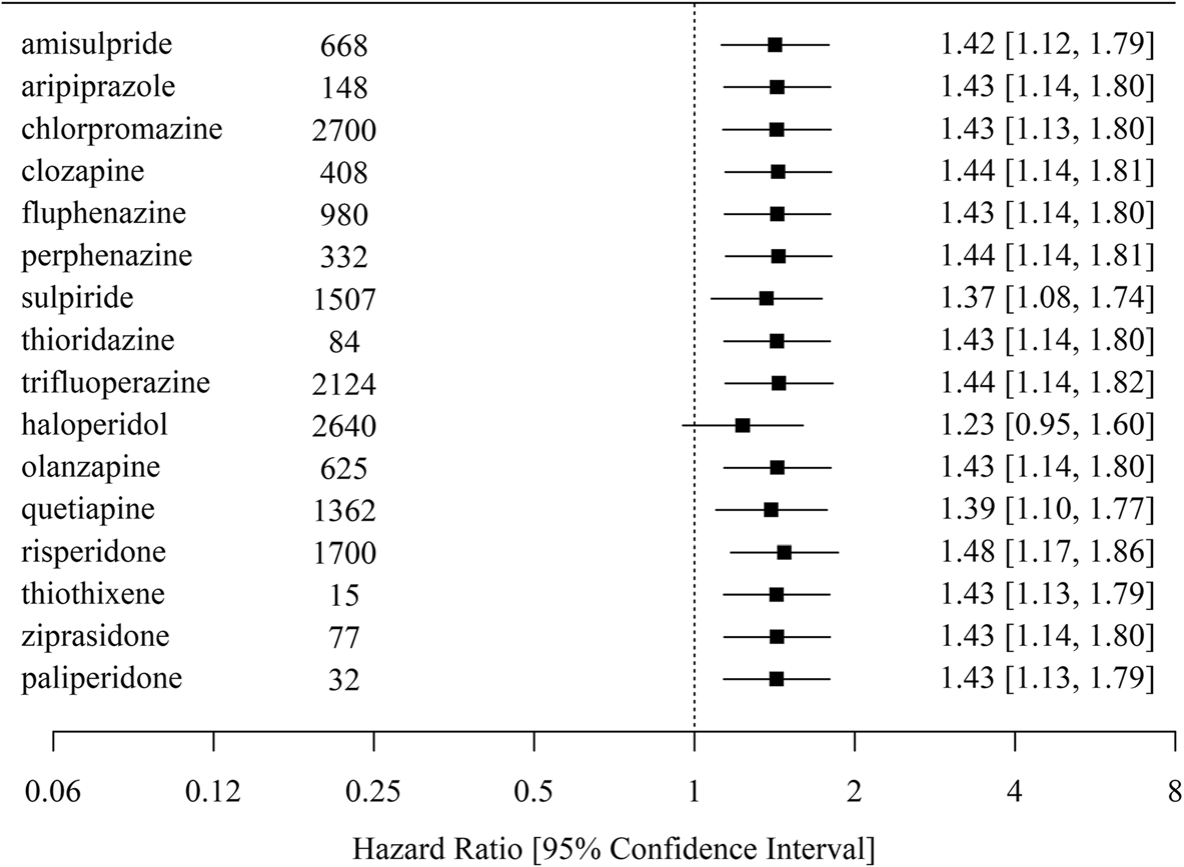
Forest plot showing the hazard ratios of ischemic heart disease for antipsychotic use among women from the replicated main analysis with each of the listed 16 antipsychotics omitted. The number of patients in the cohort who were prescribed the antipsychotic at least once in the past 12 months is also shown

As shown in Fig. 3, in the 16 replications of the main analysis with each of the antipsychotic medications omitted in defining antipsychotic use, the hazard ratios for antipsychotic use among women remained similar, except for the analysis with haloperidol omitted: the association between antipsychotic use and IHD attenuated and became non-significant (HR = 1.23, 95% CI 0.95–1.60).

When haloperidol was adopted as the main exposure, the hazard ratio for antipsychotic use among women was 2.51 (95% CI 1.65–3.79).

Among those prescribed antipsychotics at least once and each specific antipsychotic medication included as an independent variable, no interaction between antipsychotics and sex or main effects of antipsychotic use were significant (*P* > 0.05).

Additional file 1 Table S1 shows the hazard ratios for antipsychotic use among women with each specific type of acute IHD omitted in defining the outcome. The strength of association remained largely consistent with the omission of the eight specific types of IHD (HR ranged from 1.37 to 1.49).

With the threshold of the number of antipsychotic prescriptions adopted to define antipsychotic use varying from one to eight, Additional file 2: Table S2 shows the hazard ratios for antipsychotic use among women estimated from the sex-combined model (with interaction between antipsychotic use and sex). The strength of association remained similar across this range of thresholds (HR ranged from 1.39 to 1.64).

The replicated analysis on a sample selected without applying the exclusion criteria of previous hospital admission and IHD diagnosis showed that the hazard ratio for antipsychotic use among women was estimated at 1.43 (95% CI 1.24–1.65), which was the same as the main analysis but with a narrower confidence interval.

The analysis replicated with diabetes, hypertension, lipid disorder, and statin prescription excluded as potential confounders also provided highly similar results with the main analysis, with the hazard ratio for antipsychotic use among women estimated at 1.38 (95% CI 1.10–1.73).

## Discussion

In this study, we identified a higher risk of acute IHD among Hong Kong Chinese middle-aged and older primary care recipients on antipsychotics, and this heightened risk was only evident among women but not among men. Sensitivity analyses also suggested that a specific antipsychotic medication, i.e., haloperidol, may explain this elevated risk in women.

### Relationship with the literature

To the best of our knowledge, this is the largest study on the sex difference of the association between antipsychotics and IHD incidence in a primary care population. Our results are consistent with a previous study of 277,817 adults which showed a more substantial elevation of the risk of IHD among women with various psychiatric disorders treated with antipsychotics compared with men [16], but are inconsistent with a meta-analysis which showed that the association was slightly stronger among men than among women in a pooled analysis [5]. This inconsistent finding may have been due to the different patient populations adopted by the included studies. In fact, most reviewed studies only included patients with certain psychiatric disorders, such as schizophrenia, unlike our current study which observed a much more diverse population of primary care patients [5].

The sensitivity analysis of the current study extends this result to suggest that a specific antipsychotic medication, i.e., haloperidol, rather than antipsychotics in general, may underlie this sex difference.

### Interpretations

With no randomization procedures conducted, there is a risk of confounding-by-indication: the observed association between antipsychotic use and IHD may only be a consequence of an underlying factor which induced antipsychotic use and caused IHD. The observed sex moderation, nevertheless, possibly suggests potentially different mechanisms of the physiological responses to antipsychotics (or to the unobserved underlying factors which prompted antipsychotic use) and the shaping of cardiovascular risks between men and women. Our findings should inform further research hypotheses on the underlying mechanisms of the relationship between antipsychotics and IHD and the clinical practices in managing the cardiovascular risks among patients on antipsychotics.

Increased metabolic abnormalities are the most widely accepted intermediary mechanisms in previous research to explain the observed elevated risk of IHD among people on antipsychotics [17]. These abnormalities include weight gain and obesity [18], elevated glucose levels [19], and lipid disorders [20]. Animal research has suggested a potential physiological difference between male and female in the development of these abnormalities [21], with being female associated with higher risks in general. While the animal-to-human interpolation of the physiological differences is subject to further verification, epidemiological research has observed significantly higher prevalence of antipsychotic-associated metabolic abnormalities, such as elevated glucose levels [22] and weight gain among women [23]. Further research with a longer follow-up period should investigate the potential intermediary role of different metabolic condition diagnoses in the association between antipsychotic use and IHD.

Previous studies have also shown that, to achieve the same degree of psychotic symptom reduction, a lower dose of antipsychotics is typically needed for women than for men [24]. It is possible that the observed sex moderation of the association between antipsychotics and IHD was due to doses that were higher than necessary to achieve the same anticipated clinical response in women as in men. Limited by data availability, nevertheless, we did not include antipsychotic dosage and severity of psychotic symptoms in the analysis to investigate this possibility. Future research should incorporate these factors in the statistical analyses.

Another possible explanation of the sex difference of the association between antipsychotics and IHD is the different levels of medication adherence between men and women. There is research showing that men with mental disorders like schizophrenia and bipolar disorders demonstrated lower adherence to antipsychotic prescriptions than women did [25]. Hence, the observed effect modification could be a consequence of a greater proportion of men not taking their antipsychotics as instructed compared with women. Further research should include measures of medication adherence to adjust for this bias.

### Limitations

Despite consistent coding of diseases by registered physicians in the database and the large sample size with sociodemographic characteristics similar to previous primary care research in Hong Kong [26], there are several limitations to this study that require caution while interpreting the results. First, we did not implement any randomization procedures to isolate the independent effect of antipsychotics. Results should be interpreted as possibly confounded by the factors which are conducive to the prescription of antipsychotics, such as severity of the condition. Second, the moderate effect size of the association and the lack of evidence of a dose-response relationship between antipsychotic use and IHD should be taken into consideration while inferring about causality. Third, the coding of psychiatric disorders in the data is scarce and likely underestimates the true prevalence judging by the wide prescription of antipsychotics typically for treating those disorders. This problem renders the delineation of the respective independent effects of antipsychotics and the disorders infeasible. Fourth, we did not have access to lifestyle and more detailed sociodemographic information of the patients to capture the potential behavioral pathways of the investigated association. Likewise, no intermediary biometric parameters such as weight gain or blood glucose levels were available for an examination of the potential underlying metabolic mechanisms. Fifth, we only had access to the data from the public health care sector. Patients who visited private clinics or admitted to private hospitals were not recorded. However, the public sector constitutes about 90% of the total inpatient services in Hong Kong [27] and general outpatients of public clinics are unlikely to be admitted to private hospitals. In addition, ambulances, which are publicly funded, only take patients to the Accident and Emergency Units of public hospitals.

### Implications

If substantiated by further research with a more rigorous design, our study results and the generated hypothesis may have the potential to inform primary care physicians’ practices in their management of cardiovascular risks among patients on antipsychotics. Our results also suggest that the prescription of haloperidol, which may possibly underlie the association between antipsychotics and IHD, warrants further investigation. In fact, consistent with our own findings, there has already been previous research showing a higher risk of adverse events, including cardiovascular mortality, associated with the use of haloperidol [28, 29].

## Conclusion

In conclusion, we found a moderate association between antipsychotics and IHD among female but not among male primary care patients in Hong Kong. As we lack further data on the potential intermediary variables to infer about the causal relationships between the included factors, further research should observe and capture the potential intermediary mechanisms and the dose-response relationship of this association to provide more rigorous evidence to inform clinical practices.

## Supplementary information

**Additional file 1 : Table S1.** Adjusted hazard ratios [95% confidence intervals] of ischemic heart disease for antipsychotics use among women estimated from mixed effects Cox models with each type of ischemic heart disease omitted in the operationalization*.*

**Additional file 2 : Table S2.** Adjusted hazard ratios [95% confidence intervals] of ischemic heart disease for antipsychotic use among women with the threshold of the number of antipsychotic prescriptions for the operationalization of antipsychotic use varying from one to eight*.*

## Data Availability

The data that support the findings of this study are available from the Hospital Authority of Hong Kong, but restrictions apply to the availability of these data, which were used under license for the current study, and so are not publicly available.

## Abbreviations

CI: Confidence interval
HA: Hospital Authority
HR: Hazard ratio
ICD-9: International Classification of Diseases, Ninth Revision
ICPC: International Classification of Primary Care
IHD: Ischemic heart disease
