# Supplementary material for: Association between antipsychotic use and acute ischemic heart disease in women but not in men: a retrospective cohort study of over one million primary care patients
Source: BMC Med. 2020 Nov 2;18:289. doi: 10.1186/s12916-020-01765-w (PMC7604971; doi:10.1186/s12916-020-01765-w)
Supplement: Supplementary file 1 — Additional file 1 : Table S1. Adjusted hazard ratios [95% confidence intervals] of ischemic heart disease for antipsychotics use among women estimated from mixed effects Cox models with each type of ischemic heart disease omitted in the operationalization. [file 12916_2020_1765_MOESM1_ESM.docx]

| **Table S1.** Adjusted hazard ratios [95% confidence intervals] of ischemic heart disease for antipsychotics use among women estimated from mixed effects Cox models with each type of ischemic heart disease omitted in the operationalization | | | |
| --- | --- | --- | --- |
| ICD-9 | Omitted type of ischemic heart disease | n | Hazard ratio [95% confidence interval] |
| 410.01 | Acute myocardial infarction of anterolateral wall, initial episode of care | 59 | 1.41 [1.12, 1.78] |
| 410.11 | Acute myocardial infarction of other anterior wall, initial episode of care | 1,488 | 1.47 [1.15, 1.87] |
| 410.41 | Acute myocardial infarction of other inferior wall, initial episode of care | 1,154 | 1.39 [1.09, 1.77] |
| 410.51 | Acute myocardial infarction of other lateral wall, initial episode of care | 77 | 1.44 [1.14, 1.81] |
| 410.61 | True posterior wall infarction, initial episode of care | 47 | 1.43 [1.14, 1.80] |
| 410.71 | Subendocardial infarction, initial episode of care | 2,372 | 1.37 [1.04, 1.80] |
| 410.81 | Acute myocardial infarction of other specified sites, initial episode of care | 5 | 1.43 [1.14, 1.79] |
| 410.90 | Acute myocardial infarction of unspecified site, episode of care unspecified | 1 | 1.43 [1.14, 1.79] |
| 410.91 | Acute myocardial infarction of unspecified site, initial episode of care | 578 | 1.43 [1.13, 1.82] |
| 411.0 | Postmyocardial infarction syndrome | 10 | 1.43 [1.13, 1.79] |
| 411.1 | Intermediate coronary syndrome | 2,522 | 1.49 [1.12, 1.98] |
| 411.89 | Other acute and subacute forms of ischemic heart disease, other | 29 | 1.41 [1.12, 1.78] |
