# Supplementary material for: Association between antipsychotic use and acute ischemic heart disease in women but not in men: a retrospective cohort study of over one million primary care patients
Source: BMC Med. 2020 Nov 2;18:289. doi: 10.1186/s12916-020-01765-w (PMC7604971; doi:10.1186/s12916-020-01765-w)
Supplement: Supplementary file 2 — Additional file 2 : Table S2. Adjusted hazard ratios [95% confidence intervals] of ischemic heart disease for antipsychotic use among women with the threshold of the number of antipsychotic prescriptions for the operationalization of antipsychotic use varying from one to eight. [file 12916_2020_1765_MOESM2_ESM.docx]

| **Table S2.** Adjusted hazard ratios [95% confidence intervals] of ischemic heart disease for antipsychotic use among women with the threshold of the number of antipsychotic prescriptions for the operationalization of antipsychotic use varying from one to eight | | |
| --- | --- | --- |
| Threshold | Number of patients being prescribed the corresponding number of antipsychotic medications | Hazard ratio [95% confidence interval] |
| 1 | 17,780 | 1.43 [1.13, 1.79] |
| 2 | 16,842 | 1.48 [1.17, 1.86] |
| 3 | 14,835 | 1.58 [1.24, 2.01] |
| 4 | 11,455 | 1.64 [1.24, 2.17] |
| 5 | 8,392 | 1.54 [1.08, 2.19] |
| 6 | 6,949 | 1.51 [1.00, 2.28] |
| 7 | 5,225 | 1.62 [1.00, 2.62] |
| 8 | 4,580 | 1.39 [0.79, 2.45] |
